# Cellular senescence, p21, and the path to fibrosis

**DOI:** 10.1038/s44318-024-00245-8

**Published:** 2024-09-30

**Authors:** Paolo S Turano, Utz Herbig

**Affiliations:** Center for Cell Signaling, Department of Microbiology, Biochemistry, and Molecular Genetics, New Jersey Medical School, Rutgers - The State University of New Jersey, Newark, NJ 07103 USA

**Keywords:** Cell Adhesion, Polarity & Cytoskeleton, Cell Cycle, Molecular Biology of Disease

## Abstract

A recent study shows that p21 (CDKN1A) regulates expression of extracellular matrix components by senescent cells, promoting tissue fibrosis and immune cell infiltration.

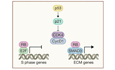

## Teaser

Senescent cells secrete bioreactive molecules that promote disease, fibrosis, and aging, yet the molecular mechanisms driving the production of these secreted factors remain incompletely understood. In this issue, Papismadov et al (2024) report that p21 (CDKN1A), known to activate the senescence growth arrest, also regulates expression of extracellular matrix components that promote fibrosis, thereby revealing new therapeutic inroads to target fibrosis and age-related pathologies.

Fibrosis is the thickening or scarring of connective tissue due to an excessive buildup of disorganized extracellular matrix (ECM) components. It is usually caused by various types of damage or chronic inflammation and can affect any organ. As fibrotic disease is a leading cause of morbidity and mortality throughout the world (Mutsaers et al, 2023), effective therapies that stop the progression towards fibrosis-induced organ failure remain an urgent need.

A main driver of fibrotic disease is cellular senescence, a state in which cells lose the ability to divide, alter their function, and secrete various pro-inflammatory and bioreactive molecules collectively called the senescence-associated secretory phenotype (SASP) (Hernandez-Segura et al, 2018). Senescent cells are abundant in fibrotic tissue and their elimination using genetic or pharmacological means in mouse models of pulmonary fibrosis results in significant improvement of physical and lung health (Hernandez-Gonzalez et al, 2023; Schafer et al, 2017; Wiley et al, 2019). As the SASP includes various pro-inflammatory cytokines and factors that can stimulate a fibrotic phenotype in fibroblasts, it has been suggested that senescent cells promote fibrosis in a non-autonomous manner due to secretion of factors that can damage and alter cells in connective tissue (Hernandez-Gonzalez et al, 2023; Schafer et al, 2017; Wiley et al, 2019). While these and other studies provide evidence that senescent cells and their secreted factors directly contribute to fibrotic disease development, the molecular mechanisms linking senescence to fibrosis, however, are still unclear.

Cells undergo senescence in response to various cell extrinsic and intrinsic stresses, such as telomere shortening and dysfunction, other types of irreparable DNA damage, oxidative stress, exposure to various cytokines, and others. These stresses ultimately activate the p53-p21 and p16-retinoblastoma (Rb) pathways and lead to the upregulation of p21 (CDKN1A) and/or p16 (CDKN2A), depending on the cell type and senescence-inducing signal. As p21 and p16 are potent inhibitors of cyclin-dependent kinases 2 (CDK2) and 4/6 (CDK4/6), both pathways inhibit CDK-mediated phosphorylation of Rb causing it to suppress the activity of E2F transcription factors and halt cell cycle progression. In senescent cells, Rb also interacts with SMAD and STAT transcription factors, thereby regulating the expression of certain SASP components (Sturmlechner et al, 2021). However, although both p21 and p16 cause hypo-phosphorylation of Rb and cell cycle arrest, the transcriptional output mediated by Rb, particularly that of SASP genes, is qualitatively different depending on which of these CDK inhibitor is upregulated in senescent cells (Chandra et al, 2022; Sturmlechner et al, 2021).

While the role of cyclin-dependent kinase inhibitors in activation and maintenance of the senescence state is well established, their contributions to fibrotic disease remain poorly understood. A new study by Papismadov et al (2024) now unravels the molecular mechanisms leading to ECM production and fibrosis by p21-expressing senescent cells. Utilizing the widely used mouse model of bleomycin (BLM)-induced pulmonary fibrosis, the authors demonstrate that fibrotic lung tissue of BLM-treated mice is highly enriched in cells expressing p21 and other senescence markers, pro-inflammatory SASP factors, ECM components, and immune cell infiltrates both 10 and 21 days after BLM administration. Such increases, however, were not observed in p21-knockout (KO) mice exposed to the same treatments, revealing that absence of p21 prior to disease onset alleviates BLM-induced development of senescent cells, inflammation, and lung fibrosis.

**Figure 1:**
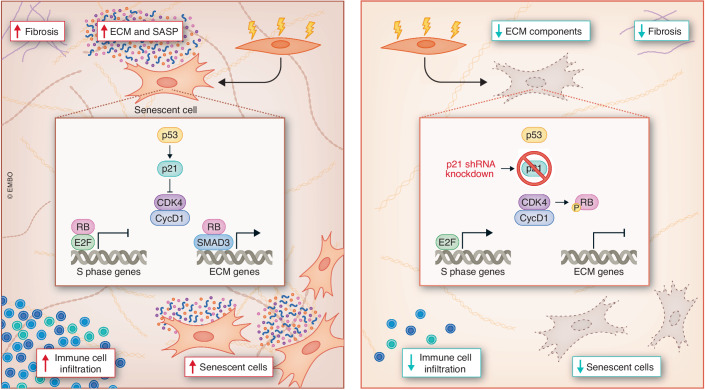
p21 regulates ECM production and promotes lung fibrosis. Left: DNA damage-induced upregulation of p21 inhibits the activity of CDK4, thereby preventing cyclin D1/Cdk4 from phosphorylating Rb. Hypo-phosphorylated Rb sequesters E2F transcription factors and prevents transactivation of genes required for S-phase entry and cell proliferation, while also promoting expression of extracellular matrix (ECM) components through SMAD3. The senescence-associated secretory phenotype (SASP) consequently contains pro-inflammatory cytokines and various ECM components that promote immune cell infiltration and fibrosis in lung tissue. Right: shRNA-mediated knockdown of p21 expression allows cyclin D1/Cdk4 to phosphorylate Rb, thereby preventing Rb from sequestering E2F transcription factors and transactivating expression of ECM components. As a result of p21 knockdown, abundance of senescent cells, ECM components, immune cell infiltrates, and fibrotic pathology is reduced. Copyright: EMBO.

Having established a central role for p21 in the pathology of lung fibrosis, the next series of experiments aimed to identify the signaling pathways mediating p21-induced ECM production and lung fibrosis. To this end, the authors first co-immunoprecipitated proteins that associate with p21 from DNA damage induced senescent (DIS) human cells and identified p21 binding partners by mass spectrometry. Unexpectedly, they discovered that CDK4, rather than CDK2, is a primary binding target of p21 in DIS cells, raising the possibility that p21 promotes the production of ECM components through a pathway involving CDK4. Indeed, while knockdown of p21 in human skin and lung fibroblasts suppressed the production of ECM components such as various collagens and fibronectin-1, combined knockdown of both p21 and CDK4 reversed these effects. Further knockdown experiments of p21, CDK4, cyclin D1, and Rb, either alone or in various combinations, followed by immunoblot and gene expression analyses revealed the involvement also of Rb and SMAD3 in ECM production of senescent fibroblasts. Importantly, the effects of p21 on ECM production were independent of the senescence state, as similar results were observed when knockdown experiments were performed in proliferating human fibroblasts. Overall, the experiments conducted in this study support the model that p21 blocks the ability of cyclin D1/CDK4 kinase to phosphorylate Rb, thereby causing hypo-phosphorylated Rb to interact with the SMAD3 transcription factor and promote the expression of ECM components in fibroblasts (Fig. 1).

While the findings in the p21 KO mouse model demonstrate a role for p21 in lung fibrosis, the complete absence of p21 prior to disease onset in this model complicates interpretation of results and prevents the assessment of targeting p21 for therapeutic applications in fibrotic disease. To address this, the authors developed a novel mouse model where p21 expression can be conditionally suppressed through doxycycline-inducible expression of a p21 shRNA in all tissues. Remarkably, shRNA-mediated suppression of p21 expression during fibrotic disease development, between days 10 and 21 after intratrachial BLM instillation, almost entirely prevented drug-induced senescent cell accumulation and ECM deposition, reduced the inflammatory response, and restored damaged architecture in lung tissue, thereby nearly completely reversing BLM-induced lung fibrosis. These data therefore demonstrate that targeting p21 after senescent cells have developed and deposited SASP components in fibrotic tissue is an effective strategy to resolve many of the pathological changes associated with lung fibrosis.

The study by Papismadov et al (2024) significantly advances our understanding of the molecular mechanisms leading to the production of ECM components in fibrotic tissue and suggests novel therapeutic targets and strategies to treat fibrotic diseases. While previous studies revealed that senescent fibroblasts promote fibrosis through secretion of factors that induce a pro-fibrotic phenotype in SASP exposed cells (Hernandez-Gonzalez et al, 2023; Schafer et al, 2017; Wiley et al, 2019), Papismadov and colleagues (2024) now demonstrate that ECM components are also being actively produced by senescent fibroblasts in a p21- and Rb-dependent manner (Fig. 1). However, since targeting p21 suppresses the production ECM components by senescent as well as by non-senescent cells, while also causing rapid cell death of p21-expressing senescent cells (Yosef et al, 2017), it is currently unclear whether the therapeutic effects of targeting p21 is primarily due to the elimination of senescent cells and their SASP, or due to suppressing synthesis of ECM components also by non-senescent cells in fibrotic tissue. In addition, as data suggest that ECM production by senescent cells is dependent on Rb hypo-phosphorylation, which is regulated by various cyclins, CDKs and CDK inhibitors, it remains to be established whether similar therapeutic effects can be accomplished by targeting other components in this pathway. Future research should focus on further dissecting the complex interplay between cellular senescence, ECM production, and fibrosis while also exploring the therapeutic potential of targeting this pathway in clinical settings.
